# Efficient biodegradation of chlorophenols in aqueous phase by magnetically immobilized aniline-degrading *Rhodococcus rhodochrous* strain

**DOI:** 10.1186/s12951-016-0158-0

**Published:** 2016-01-16

**Authors:** Jianfeng Hou, Feixia Liu, Nan Wu, Jiansong Ju, Bo Yu

**Affiliations:** CAS Key Laboratory of Microbial Physiological and Metabolic Engineering, Institute of Microbiology, Chinese Academy of Sciences, Chaoyang, 100101 Beijing People’s Republic of China; College of Life Science, Hebei Normal University, Yuhua, 050024 Shijiazhuang People’s Republic of China; University of Chinese Academy of Sciences, Shijingshan, 100049 Beijing People’s Republic of China

**Keywords:** Chlorophenols, *Rhodococcus rhodochrous*, Magnetic immobilization, Bioremediation

## Abstract

**Background:**

Chlorophenols are environmental contaminants, which are highly toxic to living beings due to their carcinogenic, mutagenic and cytotoxic properties. Bacterial degradation has been considered a cost-effective and eco-friendly method of removing chlorophenols, compared to the traditional physical–chemical processes.

**Results:**

In this study, we first developed an efficient process for the biodegradation of chlorophenols by magnetically immobilized *Rhodococcus rhodochrous* cells. *R. rhodochrous* DSM6263 degrades chlorophenols following the first step of hydroxylation at the *ortho*-positions of chlorophenolic rings. The cells immobilized by *k*-carrageenan with 9 g/L Fe_3_O_4_ nanoparticles could efficiently degrade 2-chlorophenol, 4-chlorophenol, 2,3-dichlorophenol and their mixture, which were even higher than those by free cells. The magnetically nanoparticle-immobilized cells could be used at least for six cycles.

**Conclusion:**

Given the much easier separation by an external magnetic field and high degradation efficiency, this study provides a promising technique for improving biocatalysts used in the bioremediation process for chlorophenols in wastewater.

## Background

Chlorophenols (CPs) are aromatic ring structures containing at least one chlorine atom and one hydroxyl group at the benzene rings. CPs and their derivatives are highly toxic to living beings due to their carcinogenic, mutagenic and cytotoxic properties, which have been listed as priority pollutants and potential human carcinogens by the World Health Organization and the United States Environmental Protection Agency [1].

Physical extraction and chemical oxidation have been used for the removal of CPs from wastewater, while they are expensive and not eco-friendly [2, 3]. Conversely, biodegradation of CPs has gained much attention due to its effective and eco-friendly process, since CPs are completely mineralized by microorganisms in the environment [4, 5]. CPs are subject to both anaerobic and aerobic metabolism. Under anaerobic conditions, CPs can undergo reductive dechlorination when suitable electron-donating substrates are available [6]. Aerobic degradation of CPs and their derivatives have been extensively investigated in bacteria. Recently, Arora and Bae [1] have well reviewed the biodegradation mechanism of CPs. In aerobic metabolic pathway, monooxygenases may catalyze hydroxylation at the *ortho*-positions of the chlorophenolic rings, which results in the formation of chlorocatechols that may be degraded further via *ortho*- or *meta*-cleavage of the aromatic ring, which is then completely mineralized.

Additionally, the use of immobilized microorganisms rather than free cells in biotransformation is advantageous to enhance the stability of the biocatalyst and to facilitate its recovery and reuse. And immobilization could also protect the cells and thus increase the tolerance to high concentration of pollutants. These advantages have encouraged researchers to investigate the application of immobilized cells in the biodegradation of toxic compounds [7, 8]. Several means of immobilizing cells are available, including surface adsorption, natural or artificial flocculation, covalent or electrostatic binding to carriers, and encapsulation in a polymer-gel [9–11]. However, mass transfer limitation involved in substrate diffusion to the reaction system is still the major drawback in the application of an entrapment technique. Nanoparticles (NPs) represent a new generation of environmental remediation technologies that could provide cost-effective solutions to some of the most challenging environmental clean-up problems [12]. The magnetic materials are widely used in various fields, such as drug delivery and biosensing, environmental remediation and biotechnological production process [13–16]. Therefore, the exploitation of nanoscale technology in environmental applications appears very promising. However, to date, using magnetic nanoparticle-immobilized cells to degrade CPs has not been reported.

Furthermore, the rhodococci are a group of Gram-positive bacteria that have useful industrial and/or ecological applications due to their diverse range of metabolic activities. Some rhodococci can degrade various aliphatic and aromatic hydrocarbons, making these organisms ideal candidates for use in white biotechnology and/or bioremediation [17]. In this study, we first demonstrated a new process for CPs biodegradation employing magnetically immobilized *Rhodococcus rhodochrous* cells. The high degradation efficiency and easy separation process provides a promising technique for bioremediation of CPs in aqueous phase.

## Results

### Biodegradation of chlorophenols by *R. rhodochrous* DSM6263

**Fig. 1:**
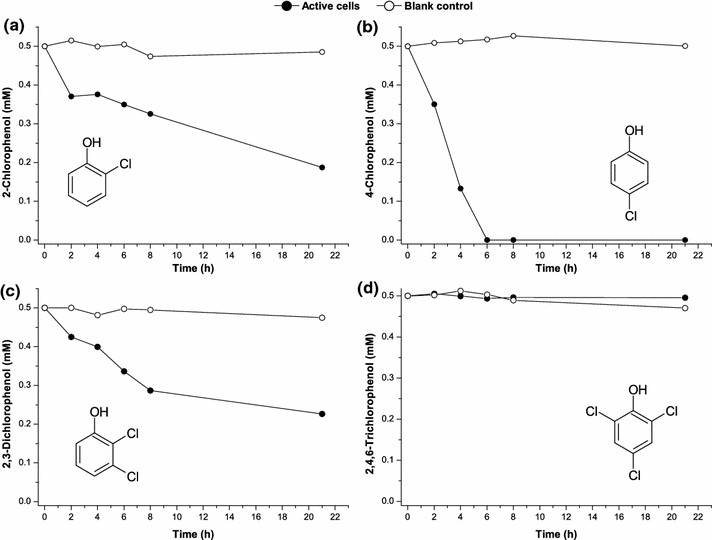
The degradation of different chlorophenols by free resting cells of *Rhodococcus rhodochrous* DSM6263. The substrates used were **a** 2-chlorophenol, **b** 4-chlorophenol, **c** 2,3-dichlorophenol and **d** 2,4,6-trichlorophenol, respectively. The initial chlorophenol concentrations were 0.5 mM. The medium without strain inoculation was used as blank control to exclude any possible photodegradation

*R. rhodochrous* DSM6263 has been isolated as an aniline and monochloroaniline degrader [18]. It metabolized aniline exclusively via the β-ketoadipate pathway (with catechol as the central metabolite) while failed to metabolize monochlorinated anilines in the absence of additional carbon sources. *R. rhodochrous* DSM6263 was also reported to be capable of growing with phenol as well as benzoate as the sole carbon and energy source [19]. Then it is of interests to investigate if the strain could also degrade CPs. 2-CP, 4-CP, 2,3-dichlorophenol (2,3-DiCP) and 2,4,6-trichlorophenol (2,4,6-TriCP) were chosen as typical CPs respectively to test the strain’s degradation capacities by the resting cells. As shown in Fig. 1, the degradation rate of 4-CP was the most fastest while the degradation behavior of 2-CP and 2,3-DiCP was slower than that of 4-CP. *R. rhodochrous* DSM6263 could efficiently degrade 0.5 mM 4-CP in 6 h while only about 0.25 mM 2-CP or 2,3-DiCP was degraded in 22 h. This phenomenon indicated that CPs degradation by *R. rhodochrous* DSM6263 should follow the first step of hydroxylation at the *ortho*-positions of chlorophenolic rings, which results in the formation of chlorocatechols. No observation of degradation for 2,4,6-TriCP further supported the above speculation since the two *ortho*-positions have been occupied by chlorine atoms, making *R. rhodochrous* DSM6263 could not attack 2,4,6-TriCP anymore. Interestingly, *R. rhodochrous* DSM6263 degraded 2,3-DiCP a little faster than 2-CP. The values of lethal concentration 50 (LC50) for *R. rhodochrous* DSM6263 with CPs were 1.0 mM for 2-CP, 1.5 mM for 4-CP and 0.5 mM for 2,3-DiCP, respectively.

### Selection of suitable immobilization supports

**Fig. 2:**
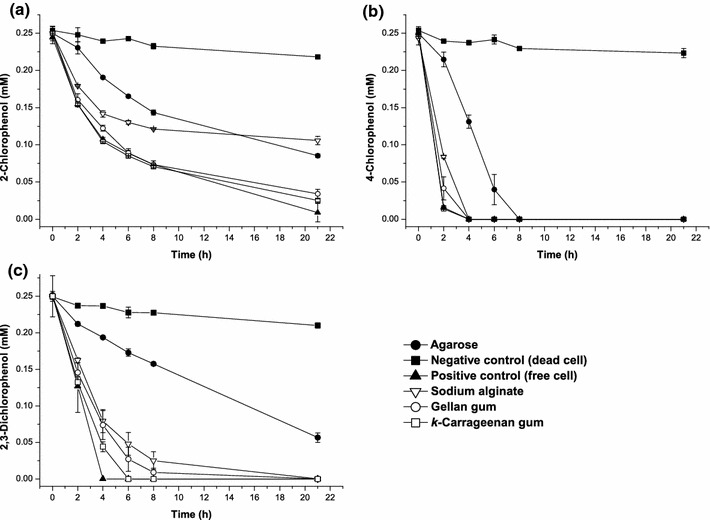
Biodegradation of chlorophenols by immobilized cells of *Rhodococcus rhodochrous* DSM6263 with different immobilization supports. **a** 2-chlorophenol, **b** 4-chlorophenol and **c** 2,3-dichlorophenol. The initial chlorophenol concentrations were 0.25 mM. The free active cells and inactive cells (autoclaved at 115 °C for 5 min) were used as positive and negative controls, respectively. The *error bars* in the figure indicate the standard deviation of three parallel replicates

Although CPs degradation by the free cell system has been demonstrated [6], the use of immobilized microorganisms rather than free cells in biotransformation is advantageous to enhance the stability of biocatalyst, which is an efficient way to reduce the costs [14]. Then different supporting materials, including gellan gel, sodium alginate, agarose and *k*-carrageenan, were selected for immobilization of *R. rhodochrous* DSM6263 cells in this study since these supports have good mechanical, chemical, thermal, and biological stability [9]. The biotransformation results indicated *k*-carrageenan was the best support since it favored all the tested CPs degradation. The *k*-carrageenan immobilized cells could degrade 2-CP and 4-CP as fast as that obtained by using free cells, although the degradation rate of 2,3-DiCP was slightly slower than that of free cells (Fig. 2). Additionally, no significant decrease of CPs content was observed when *k*-carrageenan without cells served as biocatalysts (data not shown), which further confirmed that the removal of CPs was not due to adsorption but to biodegradation by *R. rhodochrous* DSM6263 cells.

### Optimization of Fe_3_O_4_ NPs concentration for immobilization support

**Fig. 3:**
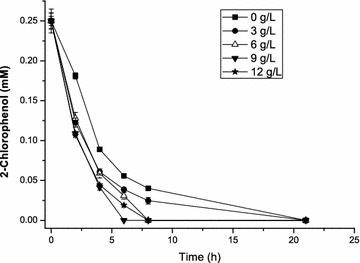
Optimization of Fe_3_O_4_ NPs concentration in immobilization support. *k*-Carrageenan was used as the immobilization support. The effect of varying the Fe_3_O_4_ NPs concentration (0, 3, 6, 9, and 12 g/L) was investigated. The *error bars* in the figure indicate the standard deviation of three parallel replicates

The magnetic Fe_3_O_4_ NPs, as part of the magnetic immobilization support, have a large surface area and are superparamagnetic [13], [14]. Therefore, the concentration of Fe_3_O_4_ NPs was expected to influence CPs degradation. 2-CP was chosen as the model substrate since its degradation rate was the slowest among all the tested CPs. Figure 3 shows the effect of different concentrations of Fe_3_O_4_ NPs on the degradation rate by immobilized cells, and 9 g/L Fe_3_O_4_ NPs was shown to be the most effective. The magnetically immobilized cells could completely degrade 0.25 mM 2-CP in 6 h with a 90 % depletion rate within 4 h, which was much higher than that of cell immobilization without Fe_3_O_4_ NPs.

**Fig. 4:**
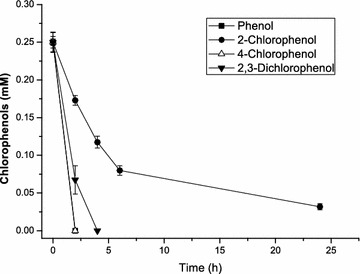
Biodegradation of phenol and chlorophenol mixture by magnetic immobilized cells of *Rhodococcus rhodochrous* DSM6263. *k*-Carrageenan and 9 g/L Fe_3_O_4_ NPs were used as the immobilization support. The mixture contained phenol, 2-chlorophenol, 4-chlorophenol and 2,3-dichlorophenol. Each compound was added at the initial concentration of 0.25 mM, respectively. The *error bars* in the figure indicate the standard deviation of three parallel replicates

After establishing an efficient immobilization process for 2-CP degradation, we further tested the efficiency for other CPs degradation. Then a mixture of phenol, 2-CP, 4-CP and 2,3-DiCP was used as substrate to model the CPs wastewater treatment. Each compound was set at an initial concentration of 0.25 mM with a total phenol concentration of 1.0 mM. As shown in Fig. 4, the magnetically immobilized *R. rhodochrous* DSM6263 cells could efficiently degrade phenol and all the CPs simultaneously. Phenol and 4-CP was depleted within 2 h and 2,3-DiCP could be consumed within 4 h. Notably, even the degradation of 2-CP was much prolonged perhaps due to the substrate inhibition under such high CPs concentration, 0.17 mM 2-CP was also simultaneously degraded within 6 h in the system, still indicated the feasibility of the immobilized process for application in CPs wastewater treatment.

### Repeated use of magnetically immobilized cells for 2-CP biodegradation

**Fig. 5:**
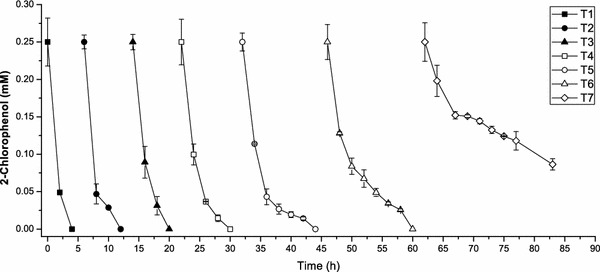
Repeated use of magnetically immobilized cells of *Rhodococcus rhodochrous* DSM6263 for 2-chlorophenol degradation. *k*-Carrageenan and 9 g/L Fe_3_O_4_ NPs were used as the immobilization support. T1 to T7 mean the recycle times. The *error bars* in the figure indicate the standard deviation of three parallel replicates

The activities of magnetically immobilized cells (at the optimal Fe_3_O_4_ nanoparticle content of 9 g/L) were tested repeatedly. As shown in Fig. 5, from the first to the sixth cycle, a total of 1.5 mM 2-CP was completely consumed by magnetically immobilized cells of *R. rhodochrous* DSM6263. The biodegradation capacity was decreased significantly in the seventh cycle due to the high gel bead broken ratio. That mean the magnetically immobilized cells could be used at least for six cycles.

## Discussion

*R. rhodochrous* DSM6263 was previously reported to degrade all the unsubstituted aromatic compounds by the action of inducible enzymes and all of the monochlorinated derivatives of aniline, phenol and benzoate, respectively, failed to support cell growth of the organisms [19]. In this study, *R. rhodochrous* DSM6263 was proven to metabolize CPs following the first step of hydroxylation at the *ortho*-positions of chlorophenolic rings, which results in the formation of chlorocatechols. The strain could degrade CPs via the constitutively expressed enzymes since the cells were cultivated in the rich medium (M65), prior to make the resting cells. This phenomenon sounds like to be consistent with another aniline-assimilating bacterium *Rhodococcus* sp. AN-22 [20], while *cis, cis*-muconic acid, the subsequently metabolic product from catechol, was unexpected to be non-metabolizable by resting cells of *R. rhodochrous* DSM6263 [19]. *R. rhodochrous* DSM6263 could degrade aniline and phenol to catechol via the β-ketoadipate pathway but could not metabolize the further metabolite, *cis, cis*-muconic acid from most of the phenol metabolism pathway. This means the degradation mechanisms between *Rhodococcus* sp. AN-22 and *R. rhodochrous* DSM6263 are different. The details of biodegradation mechanism in strain *R. rhodochrous* DSM6263 need to be further investigated.

Additionally, although CPs degradation by the free cell system has been well demonstrated [6], the biodegradation of CPs by immobilized cells was rare reported. Cells immobilization has been proven to be an effective process for removal of pesticide, which are more efficient than freely suspended cells through in situ or ex situ remediation techniques [21–23]. The white-rot fungus *Anthracophyllum discolor* immobilized on wheat grains was evaluated for CPs degradation in allophanic soil columns activated by acidification. The CPs were removed efficiently in soil columns by both adsorption and degradation processes [24]. Lee et al. [25] also immobilized catechol 1,2-dioxygenase onto a natural enzyme support, fulvic acid-activated montmorillonite. The immobilized enzyme exhibited notably increased stability against changes in the surrounding environment, such as temperature, pH, and ionic strength. In this study, the *k*-carrageenan immobilized cells could efficiently degrade various CPs as fast as that obtained by using free cells. *k*-Carrageenan has a loosen inner environment which might explain its most suitability for material transport and therefore, increased the degradation rate of CPs in this study.

Notably, NPs represent a new generation of biotransformation technology that could provide cost-effective solutions, which is advantageous by the two following factors. Firstly, their size (1–100 nm), characteristic of NPs, allows for large surface area and high specific energy. Secondly, their flexibility makes them versatile for use in both in situ and ex situ environments [14]. There have been reports that the remediation of Cr(VI) and Pb(II) was carried out by nanoscale zero-valent iron [26] and that engineered polymeric NPs were used in remediation of soil contaminated with PAHs [27]. However, the CPs degradation by magnetic immobilized cells was seldom reported. Chen et al. [28] reported using nitrogen-doped TiO_2_ NPs for immobilization of *Phanerochaete chrysosporium* to simultaneously remove Cd (II) and 2,4-DiCP from wastewater. In this study, the *R. rhodochrous* cells immobilized in *k*-carrageenan with Fe_3_O_4_ NPs could efficiently degrade 2-CP, 4-CP, 2,3-DiCP as well as their mixture, which were even higher than those of free cells. With the increase of Fe_3_O_4_ NPs, the biodegradation rate by immobilized cells was increased accordingly till to the Fe_3_O_4_ NPs concentration of 9 g/L. These results revealed that the biodegradation activity of the immobilized *R. rhodochrous* DSM6263 cells was significantly enhanced by adding Fe_3_O_4_ NPs, which should be beneficial to the reduced mass transfer resistance and the accelerated material transportation as compared to the traditional immobilization methods [16].

In an industrial bioremediation process, the recycling of the biocatalysts could be an important factor that determines the effectiveness of degradation over time [13]. The fungus *Phanerochaete chrysosporium* was immobilized in ca-alginate. The Ca-alginate immobilized fungal biocatalysts could be reused in five cycles without significant loss of adsorption efficiency [29]. In this study, we developed an efficient process for the degradation of CPs. The magnetically immobilized cells could be used at least for six cycles. The presence of free cells in solution presents a challenge for the downstream process as it is also necessary to quickly separate the cells from the system. Additionally, as the magnetic properties, the biocatalysts will be easily collected from aqueous phase by an external magnetic field. Thus the facilitation of recovery and stability makes the process more feasible and cost-effective. Given the much easier separation by an external magnetic field and high degradation efficiency, this study provides a promising technique for improving biocatalysts used in the bioremediation process for CPs in wastewater.

## Conclusions

In summary, we first developed an efficient magnetic cell-entrapment immobilization method for CPs biodegradation in wastewater. The magnetic immobilization process by *k*-carrageenan with Fe_3_O_4_ NPs not only reduced the mass transfer resistance of traditional immobilization processes, but also facilitated the recovery of immobilized cells in the reuse processes, thus significantly increased the economical competitiveness of this process. These results provide a promising technique for CPs wastewater bioremediation process.

## Methods

### Bacterial strain and culture conditions

*Rhodococcus rhodochrous* DSM6263 was purchased from Deutsche Sammlung von Mikroorganismen und Zellkulturen GmbH (DSMZ). The broth for cultivation (M65) contained 4 g/L yeast extract, 4 g/L glucose and 10 g/L malt extract. The culture temperature was 28 °C and initial pH was set at 7.2 [18]. All chemicals used in this study were of analytical grade and commercially available.

### Degradation of chlorophenols by free cells of *R. rhodochrous* DSM6263

The *R. rhodochrous* DSM6263 were cultivated in M65 broth to reach an OD_600_ value of 1.5. Then the cells were collected by centrifugation and washed with PBS (pH 7.2) twice to prepare the resting cells solution (OD_600_ 30). All experiments were conducted in 50-ml flasks containing 5 ml PBS with 0.5 mM substrate include phenol, 2-chlorophenol, 4-chlorophenol, 2,3-dichlorophenol and 2,4,6-trichlorophenol at 28 °C and on a reciprocal shaker at 180 rpm. The inactive cells sterilized at 115 °C for 5 min were used as control to exclude any possibility of photodegradation. The lethal concentration 50 (LC50) was defined as the respective half concentration (mM) of CPs which completely inhibited the biodegradation. The residual CPs contents in the broth were determined by HPLC.

### Preparation of non-magnetically and magnetically immobilized cells

Cells for immobilization were harvested after 24 h cultivation by centrifugation (4000 rpm, 10 min), and washed twice in PBS (pH 7.2). The immobilized supports were dissolved by heating. Agarose and *k*-carrageenan (2 % [wt/vol]) were mixed with cell suspension (OD_600_ 300) in a ratio of 3 of wet cell weight to dry agarose or *k*-carrageenan powder (wt/wt) after cooling to about 50 °C, respectively. The mixture was cut into uniform-sized particles and allowed to solidify in 3 % KCl for 2 h. The mixture of gellan gel (1 %) or sodium alginate (2 %) and cell suspension was extruded into 2 % CaCl_2_ and solidified for 2 h to prepare the non-magnetically immobilized cells. For preparation of magnetically immobilized cells, Fe_3_O_4_ nanoparticles (NPs) suspension was added to the above dissolved *k*-carrageenan with the same amount of cell suspension, and solidified in the same procedure [13]. The Fe_3_O_4_ NPs were prepared by the modified coprecipitation method as previous report [16].

### Biotransformation of chlorophenols by cells immobilized in different materials

After solidification, the beads were washed several times to remove the surface ions [11]. The immobilized cells were placed into the reaction system containing respective CPs at the initial concentration of 0.25 mM and PBS buffer (pH 7.2) and incubated at 28 °C for 21–24 h. All experiments were conducted in 100-ml flasks containing 10 ml PBS with 14 g wet weight immobilized biocatalyst on a reciprocal shaker at 180 rpm. The effects of varying bioconversion conditions, such as different immobilized supports (*k*-carrageenan, gellan gel, sodium alginate and agarose) and varying concentrations of Fe_3_O_4_ NPs (0, 3, 6, 9, 12, and 15 g/L) were investigated. The free active and inactive cells were used as positive and negative controls, respectively.

### Repeated use of magnetically immobilized cells

In the recycling experiments, after each biodegradation batch, the magnetically immobilized cells were collected by application of a magnetic field and then washed twice with deionized water to remove the free cells. And then 15 ml medium M65 was added to reactivate for 0.5 h. Then the cells were washed twice with deionized water again to remove the possible free cells and M65 medium broth. The 15 ml of fresh PBS containing 0.25 mM 2-CP were added to repeat the cycles. The amount of cells (the cell dry weight was 0.5 g) was used in all the cycle of repeated experiments. All experiments were performed in triplicate.

### Analytical methods

**Fig. 6:**
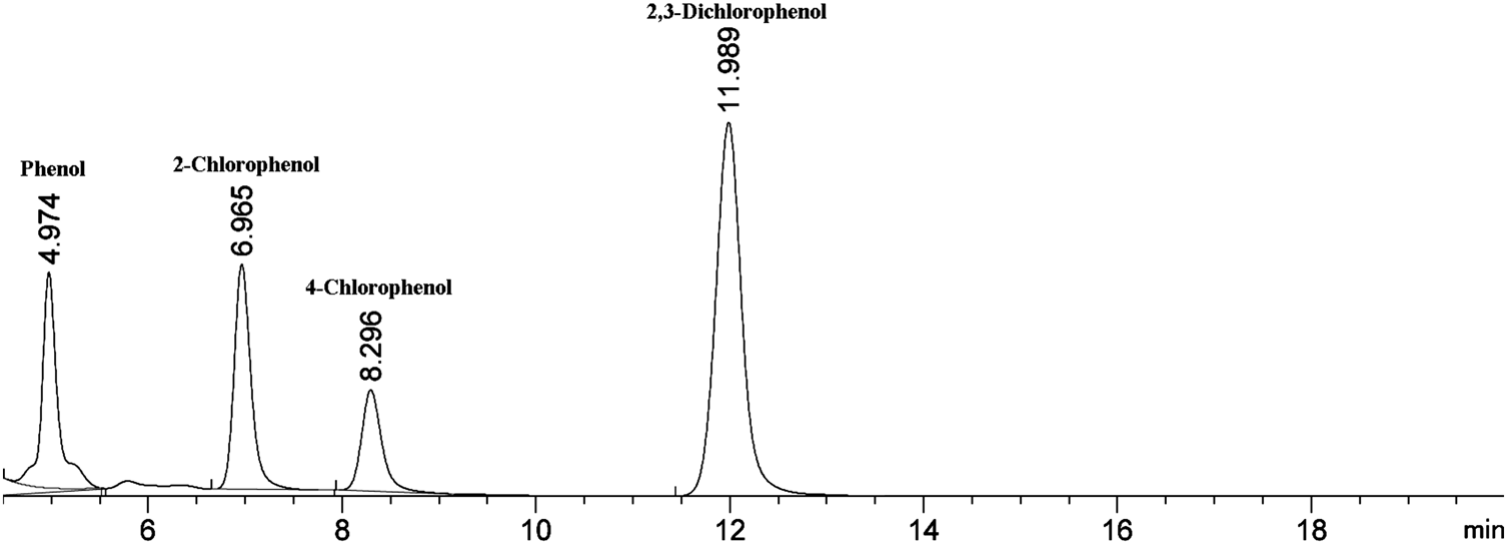
The HPLC chromatogram of different chlorophenol compounds under the same analyzing condition. The stationary phase was a reverse-phase poroshell 120 EC-C18 column. The mobile phase was a mixture of methanol and MillQ water (60:40 [vol/vol]) at a flow rate of 0.8 ml min^−1^. The injection volumes for all samples were 5 μl, respectively and the CPs were monitored at 210 nm. The retention time for phenol, 2-CP, 4-CP and 2,3-DiCP were as indicated in the figure, respectively

Cell biomass was measured in terms of optical density at 600 nm. After each batch of biodegradation, the biodegradation mixture was filtered through glass wool to separate the gel beads from the supernatant. Then, the same amount of ethanol is added to the supernatant, followed by centrifugation (10,000 rpm for 5 min) and filtration. The residual CPs contents were determined using high-performance liquid chromatography performed with an Agilent 1260 infinity instrument equipped with a reverse-phase poroshell 120 EC-C18 column (4.6 × 150 mm, 2.7 μm, Agilent). The mobile phase was a mixture of methanol and MillQ water (60:40 [vol/vol]) at a flow rate of 0.8 ml min^−1^. The injection volumes for all samples were 5 μl, respectively and all the CPs were monitored at 210 nm with a variable-wavelength detector. The CPs in mixture were analyzed in one injection, in which the induvial was distinguished by the difference of its respective retention time. The CP peaks were well separated under the above analysis conditions and the retention times for phenol, 2-CP, 4-CP and 2,3-DiCP were 4.974, 6.965, 8.296 and 11.989 min, respectively (Fig. 6).
